# Using simple models to describe the kinetics of growth, glucose consumption, and monoclonal antibody formation in naive and infliximab producer CHO cells

**DOI:** 10.1007/s10616-015-9889-2

**Published:** 2015-06-20

**Authors:** Julián López-Meza, Diana Araíz-Hernández, Leydi Maribel Carrillo-Cocom, Felipe López-Pacheco, María del Refugio Rocha-Pizaña, Mario Moisés Alvarez

**Affiliations:** 1 Centro de Biotecnología-FEMSA, Tecnológico de Monterrey at Monterrey, Ave. Eugenio Garza Sada 2501 Sur, C.P. 64849 Monterrey, Nuevo León Mexico; 2 Facultad de Ingeniería Química, Universidad Autónoma de Yucatán, Periférico Norte kilómetro 33.5, C.P. 97203 Mérida, Yucatán Mexico; 3 Biomaterials Innovation Research Center, Division of Biomedical Engineering, Department of Medicine, Brigham and Women’s Hospital, Harvard Medical School, Boston, MA 02139 USA; 4 Harvard-Massachusetts Institute of Technology Division of Health Sciences and Technology, Massachusetts Institute of Technology, Cambridge, MA 02139 USA

**Keywords:** CHO, mAb, Biopharmaceuticals, Kinetics, Monod, Luedeking–Piret

## Abstract

Despite their practical and commercial relevance, there are few reports on the kinetics of growth and production of Chinese hamster ovary (CHO) cells—the most frequently used host for the industrial production of therapeutic proteins. We characterize the kinetics of cell growth, substrate consumption, and product formation in naive and monoclonal antibody (mAb) producing recombinant CHO cells. Culture experiments were performed in 125 mL shake flasks on commercial culture medium (CD Opti CHO™ Invitrogen, Carlsbad, CA, USA) diluted to different glucose concentrations (1.2–4.8 g/L). The time evolution of cell, glucose, lactic acid concentration and monoclonal antibody concentrations was monitored on a daily basis for mAb-producing cultures and their naive counterparts. The time series were differentiated to calculate the corresponding kinetic rates (r_x_ = d[X]/dt; r_s_ = d[S]/dt; r_p_ = d[mAb]/dt). Results showed that these cell lines could be modeled by Monod-like kinetics if a threshold substrate concentration value of [S]_t_ = 0.58 g/L (for recombinant cells) and [S]_t_ = 0.96 g/L (for naïve cells), below which growth is not observed, was considered. A set of values for μ_max_, and K_s_ was determined for naive and recombinant cell cultures cultured at 33 and 37 °C. The yield coefficient (Y_x/s_) was observed to be a function of substrate concentration, with values in the range of 0.27–1.08 × 10^7^ cell/mL and 0.72–2.79 × 10^6^ cells/mL for naive and recombinant cultures, respectively. The kinetics of mAb production can be described by a Luedeking–Piret model (d[mAb]/dt = αd[X]/dt + β[X]) with values of α = 7.65 × 10^−7^ µg/cell and β = 7.68 × 10^−8^ µg/cell/h for cultures conducted in batch-agitated flasks and batch and instrumented bioreactors operated in batch and fed-batch mode.

## Introduction

The number of new biopharmaceuticals currently on the market is just over 200 (Spadiut et al. 2014; Craven et al. 2013). The economic value of these pharmaceuticals continues to expand, with sales that grew from $30 billion in 2003 (Farges et al. 2008) to $100 billion by 2012, and are expected to reach $170 billion by 2014 (Spadiut et al. 2014). Among biopharmaceutical compounds, monoclonal antibodies (mAbs) are an increasingly accepted class of therapeutics, especially in the fields of oncology, immunology, and organ transplant (Elvin et al. 2013). Since their introduction in 1986, the mAbs have become the dominant product of the biotherapeutics market (Awotwe-Otoo et al. 2012; Elvin et al. 2013; Spadiut et al. 2014). The production of biopharmaceuticals, including mAbs, relies on mammalian cell culture (Dickson 2014; Craven et al. 2013; Rodrigues et al. 2012), mainly because many therapeutic proteins require complex post-translational modifications, and mammalian cells are uniquely suited to perform these operations (Craven et al. 2013; Ho et al. 2006).

Currently, Chinese hamster ovary (CHO) cells are the most commonly used mammalian host cells in the large-scale commercial production of biopharmaceuticals (Spadiut et al. 2014; Carrillo-Cocom et al. 2014). Several factors have enabled their adoption as the industry’s main production host. CHO cells are suitable for large-scale cultivation, as they grow to very high density in suspension cultures in bioreactors of up to 10,000 L (Omasa et al. 2010). They are relatively stable in the expression of heterologous genes over time (Spadiut et al. 2014; Rodrigues et al. 2012), and they are able to properly synthesize, fold, glycosylate, and secrete complex proteins in suspension culture (Ho et al. 2006; Pascoe et al. 2007), achieving titers in the range of 5–10 g/L (Elvin et al. 2013; Omasa et al. 2010).

In this contribution, we aim to describe the kinetics of growth, product production, and substrate consumption in naive and mAb producer CHO cell cultures, the warhorse in the production of biopharmaceuticals, using simple kinetic models. Our motivation is practical and simple. Kinetic modeling has proven to be useful for understanding the relationship between process variables to improve process performance indicators (maximum cell density, product titter, and productivity) (Almquist et al. 2014; Karim et al. 2003; Alvarez et al. 2010). However, the biopharmaceutical industry is a niche wherein mathematical modeling has not been widely used. Although mammalian cells have been employed for many years in the production of biotherapeutics, information related to their kinetic parameters is scarce (Zamorano et al. 2013; Xing et al. 2010). Previously used models include the Monod-like (Frame and Hu 1991a, b; Karim et al. 2003) and logistic (Henry et al. 2008) models for hybridomas; Monod and metabolic flux (Provost and Bastin 2004); and dynamic metabolic models for nonproducing CHO cells (Zamorano et al. 2013; Provost and Bastin 2004).

Despite their wide use for more than three decades in the production of recombinant therapeutics (Kumar and Singh 2014), there are limited formal reports on the kinetics of combined cell growth and protein production in CHO cell cultures: structured kinetic (Sandadi et al. 2011) and logistic models for r-CHO producing IgG (Goudar et al. 2009); Monte Carlo methodology model for CHO cells producing B1 fusion proteins (Xing et al. 2010); and logistic and regression models for a CHO cell line producing IFN-ϒ (Farges et al. 2008).

The high added value of biopharmaceuticals is probably part of the reason why mathematical modeling has not been used as frequently in biopharmaceutical mammalian cell culture applications. The focus of the biopharmaceutical industry has been on the development of high-production cell lines, culture medium optimization, and migration of process design from batch culture to fed-batch and continuous perfusion systems. However, some regulatory, economic, and market drivers are progressively shifting attention to process control and process optimization (Craven et al. 2013).

These drivers include a stricter regulatory framework for the approval of biopharmaceutical compounds (Shukla and Gottschalk 2013), more focus in a solid science-based process knowledge or quality-by-design approach (Craven et al. 2014), and process control rather than a singular focus on robustness and reproducibility (Sidoli et al. 2004; Spadiut et al. 2013). This new context of the pharmaceutical industry also demands a better understanding of the interdependence among the key variables in the process. In addition, the increasing demand for biopharmaceuticals (Meier et al. 2014; Walsh 2010), the increasing number of companies competing for international markets (Shukla and Gottschalk 2013), and the emergence of biosimilars (Kumar and Singh 2014; Patel et al. 2014) (due to the expiration of the patents issued for the first round of commercialized mAbs), have emphasized the need for optimization and better process control.

Mathematical modeling can be a useful tool for the rational design and optimization of cell culture systems (Almquist et al. 2014). The selection of the model type depends on the end purpose of the model and the trade-offs of formulation time, model complexity, and solution time as well as the nature of the available experimental data (Craven et al. 2013). The majority of the kinetic models in the literature are categorized as unstructured because they are not only the simplest methods for modeling cell culture systems, but they provide good starting points for relatively new cell systems where data are limited, such as in this study’s case. Here, we explore the use of very simple kinetic models, namely, the Monod-type model for cell growth and substrate consumption and the Luedeking–Piret model for production. These models have been widely used for other cells of industrial importance, such as bacteria (Gerlach et al. 2014; Singh and Srivastava 2014) and fungi (Gomes et al. 2014; Slininger et al. 2014), but only modestly used for mammalian cells (Craven et al. 2013, 2014; Farges et al. 2008). In industrial practice, simple models are particularly appreciated; they are easy to use in real process characterization, online monitoring, process control and process optimization scenarios (Craven et al. 2014; Gerlach et al. 2014). In addition, they are equally effective for evaluating different bioreactor designs and devising feeding strategies (Slininger et al. 2014; Henry et al. 2008).

To our knowledge, this is the first formal report describing the kinetics of growth, substrate consumption, and product formation in mAb-producing recombinant CHO cell cultures using simple models (Monod-type and Luedeking–Piret).

## Materials and methods

### Cells and culture mediums

For these experiments, we used a CHO-S clone engineered in-house (Carrillo-Cocom et al. 2014; González-Leal et al. 2011) to produce a biosimilar of the monoclonal antibody infliximab and its native counterpart (CHO-S line from Invitrogen, Carlsbad, CA, USA). The cell culture medium consisted of CD Opti CHO (Invitrogen, Carlsbad, CA, USA) supplemented with 8-mM glutamine (Invitrogen, Carlsbad, CA, USA). Subsequently, we will refer to the naive CHO cell line and the recombinant cell line derived from it as n-CHO and r-CHO respectively.

### Batch experiments in shake flasks

Batch cultures were performed using naive (n-CHO) or recombinant cells (r-CHO) in 125-mL shake flasks with 30-mL working volume. Cultures were seeded at a viable cell density of ~2 × 10^5^ cells/mL and maintained at 33 or 37 °C in an 8 % CO_2_ humidified orbital shaking incubator at 125 rpm (Sanyo, San Diego, CA, USA). Experiments were run at different initial substrate concentrations, ranging from 1.2 to 4.8 g/L of glucose (1.2, 2.4, 3.6, and 4.8 g/L). The basal medium was diluted with PBS 1× (Invitrogen, Carlsbad, CA, USA) to achieve the desired glucose concentrations. All batch cultures were performed in triplicate with daily sampling. Cultures were terminated at day 7 for the r-CHO cell line and at day 10 for the n-CHO line, which corresponded with a viability drop to below 50 %. All samples for glucose, lactic acid, and antibody concentration determinations were centrifuged at 5000 rpm for 5 min. The supernatant was collected and stored at −20 °C until analyzed.

### Experiments in instrumented bioreactors

Bioreactor-batch and fed-batch cultures were performed only with the recombinant cell line at basal medium concentration. Experiments were performed in 1.6-L bioreactors (DASGIP, Julich, Germany) with 1.0-L working volume. The fed-batch protocol consisted of a temperature reduction (31 °C) on the seventh day and five intermittent feed additions (CD EfficientFeed™ B, Invitrogen, Carlsbad, CA, USA) of 10 % the initial volume starting on day 1. Cultures were seeded at a viable cell density of ~2 × 10^5^ cells/mL and maintained at 33 °C with an 80-rpm rate of agitation. Cultures were supplied with a gas mixture (2 sL/h) consisting of air, oxygen, nitrogen, and carbon dioxide to maintain a dissolved oxygen saturation of 40 %. All cultures were performed by duplicate with daily sampling. Batch cultures were terminated after 10 days and fed-batch cultures after 20 days. Samples for glucose, lactic acid, and antibody concentration determinations were collected and centrifuged at 5000 rpm for 5 min. The supernatant was stored at −20 °C for analysis.

### Analytical methods

Cell density and viability were evaluated via the trypan blue dye exclusion assay using a flow cytometer (Guava^®^ easyCyte™ 8HT Flow Cytometry System, Millipore, Billerica, MA, USA). A 40-mW laser with a wavelength of 635 nm was used for excitation. Samples were analyzed with a particle rate of 500 particles/µL and 1000 events. Glucose and lactic acid concentrations were measured by HPLC using a Waters chromatography system (model W1515) coupled to a refractive index detector (model W2414, Waters, Milford, MA). An Aminex^®^ HPX 87H (Bio-Rad, Hercules, CA) column and a 5-mM sulfuric acid mobile phase were used at a flow rate of 0.6 mL/min and temperatures of 50 and 60 °C in the detector and oven respectively. An enzyme-linked immunosorbent assay (ELISA) was used to quantify the active monoclonal antibody in the collected samples, as described elsewhere (Carrillo-Cocom et al. 2014).

### Kinetics analysis

The viable cell [X] and glucose concentration [S] profiles observed in the exponential phase of batch experiments were fitted to second-order polynomial equations using Excel Software (Microsoft^®^, Redmond, WA, USA). Polynomial equations were derived to calculate the experimental cell production rate (**r**_x_), where r_x_ = d[X]/dt, and [X] is cell concentration; and the glucose consumption rate (**r**_s_), where rs = d[S]/dt, and [S] is glucose concentration. The rates were analyzed to determine the relationships between substrate consumption and cell concentrations; namely the cell yield coefficient (Y_x/s_).

In addition, the values of the specific growth rate at different initial substrate concentrations were generated for each cell line using conventional approaches. Briefly, a first-order growth was assumed to occur during the initial stage of exponential growth at each initial substrate concentration (Eq. 1).

1 $$ {\text{d}}[X]/{\text{dt}} =\upmu[X];\;{\text{with}}\;[X] = [X]_{\text{o}} \;{@}\;{\text{t}} = {\text{t}}_{\text{o}} = 0.0 $$

In Eq. 1, [X] is the biomass concentration at a given time, [X]_o_ is the cell concentration at an initial condition, t is the time, t_o_ is the initial time (corresponding to the beginning of the exponential growth phase), and µ is the specific growth rate with a constant value at least during the initial portion of the exponential phase. The integrated version of Eq. 1 renders Eq. 2, a straight-line model.

2 $$ \ln [X] =\upmu{\text{t}} + \ln [X]_{\text{o}} $$

Therefore, the value of the specific growth rate (µ) was calculated from the slope of the plots of ln[X] versus t at each initial substrate concentration value [S]_t_. A Monod-type equation (Eq. 3) was used to fit the values of the specific growth rate (µ) at different initial substrate concentrations.

3 $$ \upmu =\upmu_{\hbox{max} } [S]*/({\text{Ks}} + [S]*);\;{\text{with}}\;[S]* = [S]_{\text{o}} - [S]_{\text{t}} $$

A Lineweaver–Burk linearization was used to obtain values for the kinetic parameters: µ_max_ (the maximum specific growth rate), K_s_ (the concentration of substrate at which half the value of µ_max_ was observed), and the [S]_t_ (the minimum substrate concentration at which cell growth was observed). We only considered a particular linear regressions as valid if r^2^ was higher than 0.97.

The material balance for substrate consumption was assumed to be represented by Eq. 4.

4 $$ {\text{r}}_{\text{s}} = {\text{d}}[S]/{\text{dt}} = {\text{r}}_{\text{x}} /{\text{Y}}_{{{\text{x}}/{\text{s}}}} $$

In this equation, r_s_ is the global rate of glucose consumption, r_x_ is the rate of cell growth, t is time, and Y_x/s_ is a cell yield coefficient. In agreement with Eq. 4, the yield coefficient was calculated from the slopes of r_x_ versus r_s_ plots at different [S]_o_ values.

For the kinetic analysis of mAb production in shake flasks and bioreactors, the viable cell concentration profile [X] was fit to a sixth-order polynomial. The polynomial equation was derived to obtain the experimental cell production rate (**r**_x_) at each time. A Luedeking–Piret-type model (Luedeking and Piret 1959) was proposed for the modeling of antibody production (Eq. 5).

5 $$ {\text{d}}[P]/{\text{dt}} = {\text{r}}_{\text{p}} =\upalpha{\text{r}}_{\text{x}} +\upbeta[X];\quad {\text{r}}_{\text{x}} = {\text{d}}[X]/{\text{dt}} $$

Here, [P] is the mAb concentration at a given point, t is time, r_p_ is the rate of accumulation of product in time within the reaction vessel, [X] is the cell concentration at that given time, r_x_ is the rate of cell growth, and α and β are the Luedeking–Piret constraints.

Constants were set so that the observed experimental product profile in fed-batch cultures was closely approximated by the numerical integration of the Luedeking–Piret equation (see Eq. 6).

6 $$ P_{\text{n}} = P_{{{\text{n}} - 1}} + \left( {\upalpha{\text{r}}_{\text{x}} +\upbeta X} \right)* \,\Delta {\text{t}} $$

Here *P*_n_ is the antibody concentration at the present time step, P_n−1_ is the antibody concentration at the previous time step of the integration, α and β are the Luedeking–Piret constants, and Δt is the time step of numerical integration.

In addition, three independent sets of data from the batch culture experiment of the r-CHO cells conducted in Erlenmeyer flasks at 37 °C were used to adjust the Monod and Luedeking–Piret values during exponential growth at this temperature. For this purpose, the differential material balance equations for cell growth, substrate consumption, and mAb formation were numerically integrated using the values of µ_max_, K_s_, [S]_t_, Y_x/s_, and α and β determined at 33 °C as an initial guess. The parameters [S]_t_ and α were kept constant, while µ_max_, Y_x/s_, and β were varied iteratively until a good agreement between the [X], [S], and [P] experimental time series and the simulation values was achieved.

## Results and discussion

### Analysis of concentration profiles at different substrate concentrations

**Fig. 1:**
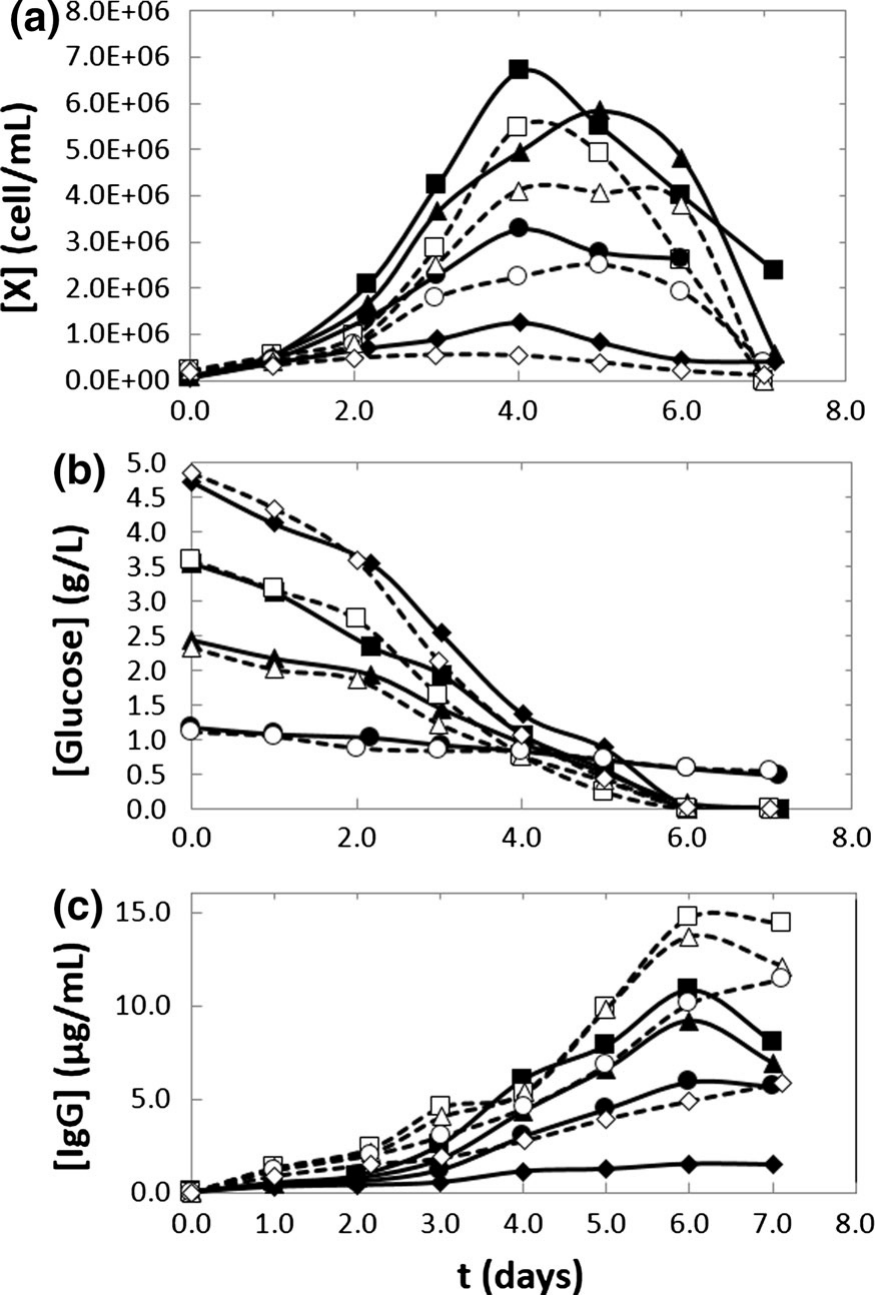
Time evolution of cell, substrate, and lactic acid concentrations for recombinant CHO cell lines cultured at two different temperatures [33 °C (*solid line with square*), and 37 °C (*dashed line with square*)], and different initial substrate concentrations 4.8, 3.6, 2.4, and 1.2 g/L (*solid line with square*, *solid line with triangle*, *solid line with circle*, *solid line with diamond*)

The culture of CHO cells in commercial culture media is a relatively well-established protocol among practitioners in biopharmaceutical industry laboratories. Normally, the culture medium is prepared according to the recommendation of the manufacturer. However, in this study, we conducted a series of batch experiments in shake flasks at different initial substrate concentrations using naive and recombinant cells to evaluate the dependence of growth kinetics with respect to substrate concentration. For that purpose, we diluted a commercial culture medium (CD Opti CHO; Invitrogen, USA). Triplicate batch culture experiments were conducted at each culture medium dilution, namely 100, 75, 50, and 25 %, with respect to the concentration recommended by the manufacturer. Our rationale was to produce relevant kinetic parameters for a commercially available and widely used culture medium (for lab scale and pilot plant applications). These dilutions approximately correspond to initial glucose concentrations of 4.8, 3.6, 2.4, and 1.2 g/L, respectively. Commonly, a bi-phase culture protocol is used to culture recombinant CHO cells. In a first culture stage, cells are culture at 37 °C to maximize growth rate. In a second stage, once the maximum cell density has been reached, the temperature is decreased to 33 or 31 °C to minimize further growth, decrease product degradation, and favor overall productivity. However, in our experiments with this particular recombinant CHO cell line, we have observed best results (in terms of overall mAb productivity) in experiments where the cell culture temperature is maintained at 33 °C throughout the entire culture period. For completeness, in this contribution, we report cell density profiles and glucose, lactic acid, and mAb concentration profiles from experiments with recombinant cells conducted at both 33 and 37 °C (Fig. 1). In general, higher maximum cell densities and mAb titters are observed at 33 C, although the glucose concentration profiles are remarkably similar. Later in this study, we provide a detail kinetic analysis of the cell growth of both naïve and recombinant cell cultures at 33 and 37 °C.

**Fig. 2:**
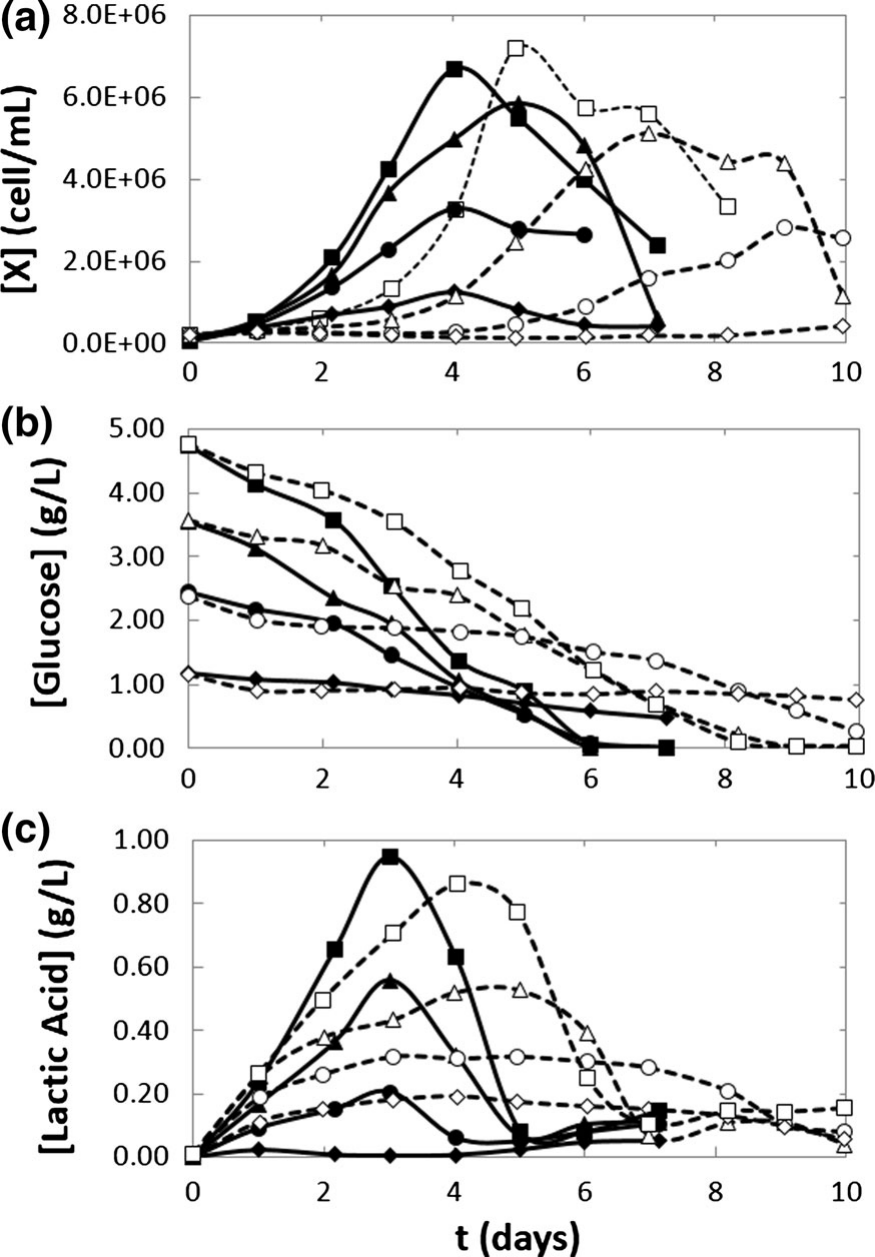
Time evolution of cell, substrate, and lactic acid concentrations for naive and recombinant CHO cell lines at different substrate concentrations. CHO-S (*dashed line with square*), r-CHO (*solid line with square*), and different initial substrate concentrations 4.8, 3.6, 2.4, and 1.2 g/L *solid line with square*, *solid line with triangle*, *solid line with circle*, *solid line with diamond*). The main difference was that the lag-phase was reduced for the r-CHO cell line; glucose consumption and lactic acid production varied similarly according to the cell concentration

Figure 2a shows the cell concentration profiles for both naive and recombinant CHO cells at 33 °C. For the experiments conducted at initial glucose concentrations of 4.8, 3.6, and 2.4 g/L, naive and recombinant cell cultures exhibited growth. Negligible cell growth was observed in experiments at a glucose concentration of 1.2 g/L. Interestingly, while r-CHO cells did not exhibit a lag-phase (even at the lowest initial glucose concentration at which growth was observed), the naive cells exhibited longer lag-phases with decreasing initial glucose concentrations. Longer lag-phases for naïve cells were consistently observed in experiments conducted at both 33 and 37 °C. At this point, we cannot provide an explanation for this behavior.

Both cell lines (naïve and recombinant) reached an average peak cell density of 7 × 10^6^ cells/mL in undiluted culture medium; no significant difference in the maximum cell density was observed between naive and recombinant cultures. For r-CHO cultures, the peak cell density was typically observed at day 4. In contrast, such consistency was not achieved on n-CHO cultures. In r-CHO cultures in undiluted medium, a sharp decline in viable cell density was observed after the peak cell density was achieved, resulting in r-CHO cell cultures lasting 2–3 fewer days than their naive counterparts. Figure 1b shows the glucose concentration profiles for naive and recombinant cultures at different culture medium dilutions. In r-CHO cultures, glucose was depleted by day 6; while for the n-CHO cultures, complete depletion occurred by day 8. Clearly, the rate of glucose consumption was significantly higher in recombinant cultures than in their naive counterparts. In all the experimental conditions tested, the rate of glucose depletion was practically time-independent from the beginning of the experiment to the complete depletion of substrate. The lactic acid concentration profiles were similar in shape to the cell growth curves, but with peak concentration generally occurring 1–2 days before the peak in cell density. A similar behavior has been observed in batch CHO cell cultures in previous studies (Amanullah et al. 2010; Goudar et al. 2005; Pascoe et al. 2007). Our experimental results suggest that in the range of the conditions tested, lactate was actively produced during the stage of exponential cell growth for later consumption once the cell density reached its peak, typical behavior recently studied in detail by Zagari et al. (2013). In our batch experiments, regardless of the initial glucose concentration, the maximum lactate concentration did not exceed 1.0 g/L.

### Specific production and consumption rates

Table 1 summarizes the rates of growth, substrate consumption, lactic acid production (or consumption), and product formation in each of the experiments run with n-CHO and r-CHO cultures conducted at 33 °C. Rates were normalized in terms of the accumulated viable cell count. In the case of glucose consumption, this analysis showed that glucose consumption rates progressively decreased (as expected) as glucose concentration decreased. Two distinct culture phases were observed in terms of substrate consumption for recombinant cultures (three for their naive counterpart). Higher consumption rates during the exponential growth phase, followed by a drastic decrease of specific consumption (more than 70 %) once the culture entered into the late exponential phase. In naive cultures, a third stage of even lower consumption was discriminated at the end of the exponential phase. Similarly, in terms of specific rates of lactose production or consumption, different metabolic stages of the culture could be distinguished (see also Tsao et al. 2005). In general, the rate of lactose production was high at the first half of the exponential phase, practically proportional to the rate of growth, but decreased significantly during the second half. This is consistent with observations from other authors (Zagari et al. 2013). After the maximum cell concentration was achieved (in our batch culture experiments, this occurred at approximately the point of glucose depletion), the accumulated lactate was used as a substrate by the cultures. Note that from day 3 (r-CHO clone) and day 4 (n-CHO cells), we observed simultaneous consumption of lactate and glucose. Interestingly, we observed that in recombinant cultures exposed to substrate-depleted conditions, the specific rate of lactate consumption was practically constant (≈0.05 ng/cell/day) and approximately five-times higher than in naive cell cultures exposed to similar glucose-depleted conditions. Our experiments also suggested that the specific rate of mAb production was higher in experiments at lower initial substrate concentrations. Remarkably, the specific production rate practically doubled its value in experiments at [S]_o_ = 1.3 g/L with respect to those conducted in undiluted medium (1.015 vs 0.58 pg/cell/day).

**Table 1:** Cell growth, glucose consumption, lactose production/consumption, and mAb production rates observed in naive (n-CHO) and recombinant (r-CHO) cells in batch cultures at 33 °C at different initial substrate concentrations. The values of these rates clearly define different metabolic phases/stages of the cultures, referred here as Phase I, II and III

[*S*]_o_ (mg/mL)	Cell line	Specific growth rate at 33 °C µ (h^−1^)	Specific glucose consumption rate q_Glu_ (ng/cell/dia)			Specific lactate production/consumption rate q_Lac_ (ng/cell/dia)					Specific production rate qmAb (pg/cell/dia)
			Phase I	Phase II	Phase III	Phase I	Phase II	Phase III	Phase IV	Phase V	
4.755	n-CHO	0.035	0.422	0.149	0.067	0.906	0.093	−0.027	−0.058	0.003	NO
3.569	n-CHO	0.032	0.289	0.171	0.064	0.593	0.076	−0.012	−0.072	−0.002	NO
2.366	n-CHO	0.022	0.195	–	–	0.278	0.004	−0.043	–	–	NO
1.154	n-CHO	0.011	0.138	–	–	–	–	–	–	–	NO
4.733	r-CHO	0.036	0.449	0.128	NO	0.376	0.105	−0.059	−0.089	0.007	0.590
3.555	r-CHO	0.035	0.324	0.086	NO	0.274	0.055	−0.055	−0.048	0.005	0.584
2.440	r-CHO	0.032	0.327	0.126	NO	0.107	0.036	−0.050		0.008	0.786
1.183	r-CHO	0.016	0.122	0.168	NO	0.089	−0.026	−0.004	0.003	0.025	1.015

–, not-determined; NO, not-observed

Overall, our results showed that growth was a strong function of substrate concentration for naive and recombinant CHO cells. With decreasing initial glucose concentration (in the window of 2–4 g/L), the rate of cell growth, the maximum cell density, and lactic acid production progressively decreased. At glucose concentrations lower than 1.2 g/L, negligible cell growth rates (and glucose consumption rates) were observed. As expected, the glucose consumption rates observed in recombinant cultures were higher than in naive ones. In the absence of glucose supplementation, both naive and recombinant cultures were able to consume lactate. Surprisingly, naive cultures exhibited a longer lag-phase and reached their highest cell density later than the recombinant cultures. The value of the maximum cell density was approximately the same for naive and recombinant cultures.

Differences related to range of growth, glucose consumption, and lactate production between naive and transformed cell clones have been reported in other studies (Ho et al. 2006; Pascoe et al. 2007). Here, we established a direct comparison in the context of mAb production of recombinant and naive cells exposed to the same experimental conditions and derived from the same commercial cell line. In the following section, the dependence of the growth rate as a function of glucose concentration will be characterized in more detail, and a Monod-type model for CHO cells will be proposed.

### A simple Monod-type kinetic model for CHO cell growth rate

The CHO cell line was developed in 1957 (Jayapal et al. 2007; Tjio and Puck 1958), and the production of monoclonal antibodies in CHO cells was reported in 1990 and 1991 (Page and Sydenham 1991; Wood et al. 1990). Surprisingly, the simple kinetic models widely used to describe the growth and production of other cells of commercial interest have not been widely used to study the dynamics of growth, production, and substrate consumption in CHO cell cultures. To further study the dependence of cell growth from substrate concentrations, we chose a Monod-type model; Monod models have a proven record as a useful model across other biological systems and scales (e.g., Craven et al. 2013). To our knowledge, there are no literature reports on the kinetic parameters from CHO cell batch culture related to mAb production.

**Fig. 3:**
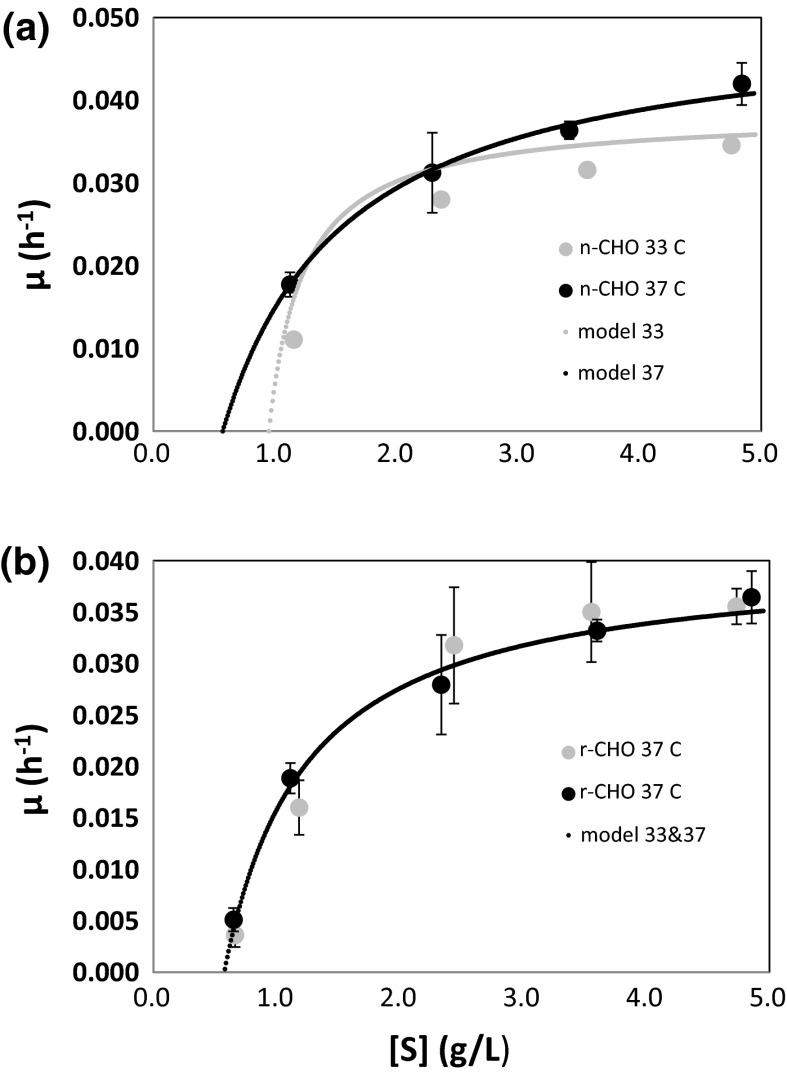
Specific growth rate at different initial substrate concentrations. For **a** naïve and **b** recombinant cell lines, a threshold substrate value of 0.6 g/L was identified. Kinetic parameters obtained via Lineweaver–Burk linearization, accounting for the threshold substrate, were µ_max_ 0.050 h^−1^ and K_s_ 2.058 g/L for the n-CHO cell line and µ_max_ 0.043 h^−1^ and K_s_ 0.929 g/L for the r-CHO cell line

From the cell growth curves in Fig. 1a, we calculated the average specific growth rate [μ (h^−1^)] during the exponential phase for every initial substrate concentration for experiments at both 33 and 37 °C. We observed that cell growth was a first-order process (Eq. 1) with respect to cell concentration, at least at the beginning of the exponential growth phase for each initial substrate concentration. We used Eq. 2, the integrated version of Eq. 1, to calculate the initial specific growth rate (µ) from the slope of the plot of ln[X] versus time constructed at each initial substrate concentration. Figure 3 shows the values of µ as a function of initial glucose concentration for naive and recombinant cell cultures. The shape of the curve suggests a Monod-type behavior. However, the curves do not originate from the zero substrate value. Therefore, a useful growth model requires the inclusion of a threshold substrate parameter. This type of growth limitation has been referred to in literature as a kinetic growth limitation (Sidoli et al. 2004; Kovarova et al. 1996). The existence of this substrate threshold has been justified based on maintenance energy and thermodynamic arguments (Kovarova et al. 1996; Ribes et al. 2004). Additional experiments were carried out with lower initial glucose concentrations, namely 0.65, 0.45, and 0.25 g/L (data not shown). Results indicated that growth stopped below 0.60 g/L.

Although the Monod model imposes the growth rate at zero only if the substrate concentration becomes zero, which was previously determined untrue for our particular cell lines, it is generally recognized that threshold substrate concentrations exist at levels below where any substrate consumption occurs (Ribes et al. 2004). Thus, the Monod model has been previously modified to account for these deviations (Frame and Hu 1991b; Kovarova et al. 1996; Ribes et al. 2004). For the present work, the model shown in Eq. 3 provided an excellent fit to our experimental data and was consistent with our experimental observation of existence of a substrate threshold for cell growth.

3 $$ \upmu =\upmu_{\hbox{max} } [S]*/({\text{Ks}} + [S]*);\;{\text{with}}\;[S]* = [S]_{\text{o}} - [S]_{\text{t}} $$

In this model, µ is the specific growth rate of the cells, µ_max_ is the maximum specific growth rate, [S]^*^ is the above-the-threshold substrate concentration, [S]_t_ is the substrate threshold concentration, and K_s_ is the half-velocity constant. The kinetic parameter values for this Monod-type model for both naive and recombinant CHO cells were obtained using a Lineweaver–Burk linearization strategy (Table 2; Fig. 3). Values of µ_max_ = 0.050 h^−1^ and K_s_ = 1.023 g/L were estimated for the naive cell cultures at 37 °C. For experiments at 33 °C, this set of parameter values changes significantly; µ_max_ = 0.038 h^−1^ and K_s_ = 0.286 g/L. The substrate threshold value to initiate cell growth also changes significantly; [S]_t_ = 0.96 g/L in experiments at 33 °C and [S]_t_ = 0.96 g/L in experiments at 37 °C. Interestingly, the Monod-type kinetic values for recombinant cells do not change significantly as a function of temperature (Fig. 3), and for all practical purposes, a single set of parameters (µ_max_ = 0.040 h^−1^; K_s_ = 0.664 g/L; [S]_t_ = 0.58 g/L) can be used to describe recombinant cell growth at 33 and 37 °C. As expected, higher specific rate values were determined for naive cell cultures at 37 °C. Intriguingly, a lower K_s_ value was observed in r-CHOs (at both 33 and 37 °C) than in n-CHOs at 37 °C, indicating that a lower substrate concentration is needed in recombinant cultures to reach at least half of the maximum rate of growth. These kinetic parameter values were consistent with the reported ranges for µ_max_ for naive CHO Cells (0.020–0.060 h^−1^) (Craven et al. 2013; Sandadi et al. 2011; Xing et al. 2010; Goudar et al. 2009; Farges et al. 2008) and slightly higher than values reported for other recombinant CHO cell lines (0.025–0.035 h^−1^) (Craven et al. 2013; Xing et al. 2010; Goudar et al. 2009; Farges et al. 2008).

**Table 2:** Kinetic parameters determined from batch experiments for naive (n-CHO) and recombinant (r-CHO) cell cultures at 33 and 37 °C

Parameter	n-CHO cells (@ 33 °C)	n-CHO cells (@ 37 °C)	r-CHO cells (@ 33 °C)	r-CHO cells (@ 37 °C)
µ_max_ (h^−1^)	0.038	0.05	0.040	0.040
K_s_ (mg/mL)	0.286	1.023	0.664	0.664
[*S*]_t_ (mg/mL)	0.96	0.58	0.58	0.58
Y_x/s_ (cell/mg)	≈6.08 × 10^6a^	≈6.08 × 10^6a^	≈2.59 × 10^6b^	1.70 × 10^6^
α (µg/cell)	0	0	7.65 × 10^−7^	7.65 × 10^−7^
β (µg/cell/h)	0	0	7.65 × 10^−8^	1.38–3.38 × 10^−8c^

^a^Value ranges from 2.69 to 10.8 × 10^6^ (see Table 2)

^b^Value ranges from 0.71 to 2.79 × 10^6^ (see Table 2)

^c^No unique value was capable of fitting experimental {t,[*X*],[*P*]} data for different sets

### Estimation of yield coefficients Y_x/s_

The cell production rate (r_x_) and the substrate consumption rate (r_s_) were calculated as the analytical derivative of second-order polynomial equations fitted to time series data of [X] and [S] during the exponential growth phase of naive and recombinant cultures. The cell/substrate yield coefficient (Y_x/s_) resulted from the reciprocal of the slope for the cell production rate versus the substrate consumption curve, see Eq. 4. Our results suggested that the cell/substrate yield coefficient was not constant, but was a function of the substrate concentration: at higher glucose concentrations, higher conversion efficiencies were observed. For the calculation of the yield coefficient of the recombinant CHO cell line, we assumed that the substrate consumption during the first 7 days of culture was devoted to cell growth; that is, no significant glucose consumption was a result of product assembly. This assumption was based on the fact that the mAb concentration at day 7 was still modest (10–15 mg/L) compared to the maximum production potential of this cell line in fed-batch cultures with supplementation (final mAb titer of 250 mg/L, data not shown). Additionally, product concentration was minimal compared to the initial substrate concentrations; 10–15 mg/L was the final mAb titer compared to 4 g/L of initial glucose. Therefore, very little substrate must have been used for product generation during the first 7 days of culture. Furthermore, by comparing the values for the yield coefficients between both cell lines (Table 3), we observed that the yield coefficients corresponding to the naive cells were significantly higher than those of the recombinant cell line. Our results suggest that in a recombinant cell, a significant portion of substrate is used to sustain protein production even at growth stages where protein expression is still not significant. In producer cells, the more complex cell metabolism associated with the synthesis capability of an exogenous protein implies higher substrate demands.

**Table 3:** Cell/substrate yield coefficient Y_x/s_ for naive (n-CHO) and recombinant (r-CHO) cell lines calculated from batch experiments conducted at 33 °C at different initial substrate concentrations

[*S*]o (mg/mL)	Y_x/s_ n-CHO-S (cells/mg)	Y _x/s_ r-CHO (cells/mg)
4.8	1.08E+7	2.79E+6
3.6	7.25E+6	3.19E+6
2.4	3.58E+6	1.81E+6
1.2	2.69E+6	7.18E+5

### Modeling of mAb production in shake flasks

**Fig. 4:**
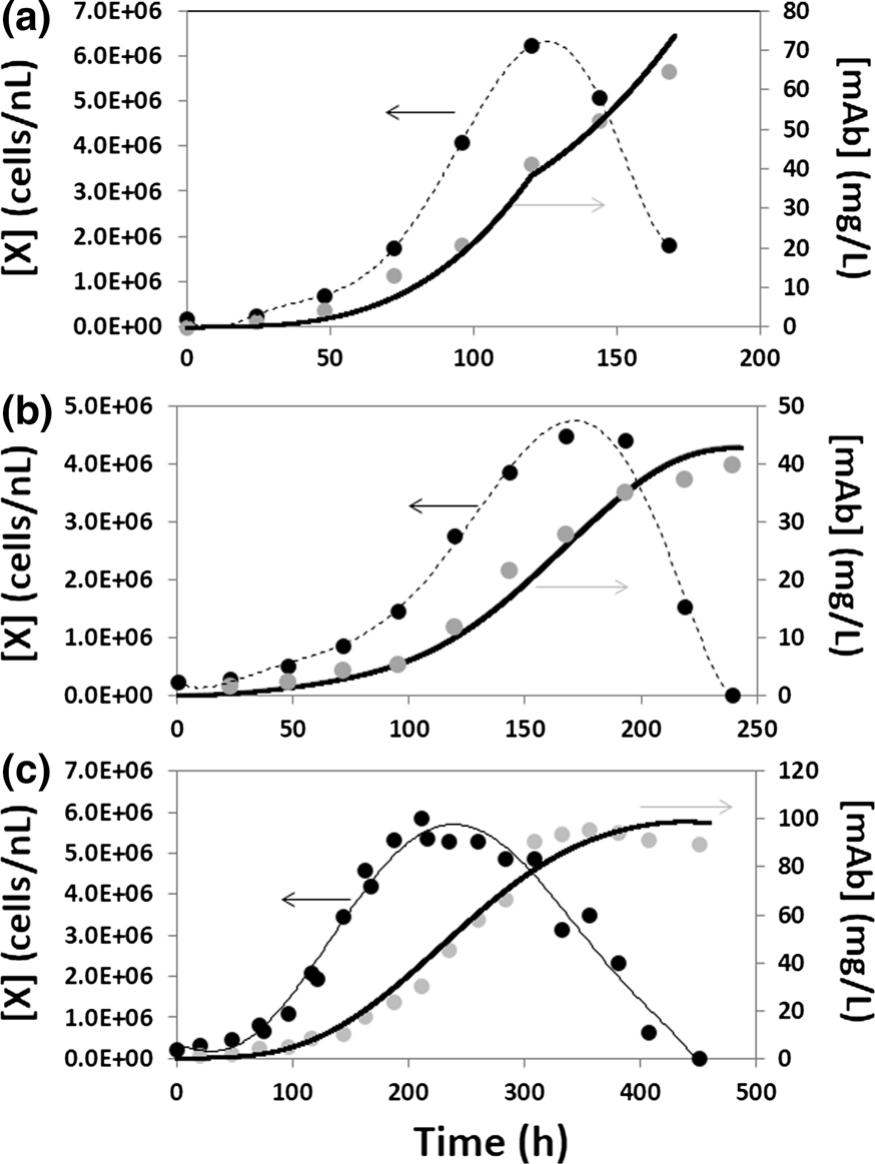
A simple Luedeking–Piret model describes the relationship between the rate of growth, the viable cell concentration, and the rate of mAb production in **a** batch experiments performed in Erlenmeyer flasks at [S]_o_ = 4.8 g/L (undiluted medium); **b** batch experiments run in 1.0-L instrumented bioreactor at [S]_o_ = 4.8 g/L; and **c** fed-batch experiments run in 1.5-L (final volume) instrumented bioreactors. Experimental viable cell data (*black dot*) was fit to a fifth-order polynomial (*dashed line*). Adequate fit (*dark line*) to the experimental mAb concentrations [mAb] (*gray dot*) was achieved using the same α,β-parameter set with α = 7.65 × 10^−7^ µg/cell/h and β = 7.68 × 10^−8^ µg/cell for all three experimental scenarios

Figure 4a shows the cell and mAb profiles for typical batch processes conducted in shake flasks. In our experiments, cultures were typically maintained for 160–190 h. On average, the maximum cell densities of 7 × 10^6^ cells/mL and final mAb titers of 60 mg/L were achieved. Typically, in mAb production processes, product accumulation continues after the cell density peaks (Templeton et al. 2013). In our batch experiments, 70 % of the final mAb titer was generated during the cell death phase of the culture.

We also studied the kinetics of the process of monoclonal antibody production and their relationship with the rate of cell growth. The production kinetics of mAb are highly cell-line dependent. Several reports have documented that antibody production is practically independent of the cell growth and substrate consumption rates (Henry et al. 2008; Sidoli et al. 2004). However, results from previous experiments with this recombinant CHO cell line suggested that it followed mixed-mode production kinetics, where at any given time the rate of product formation depends on the rate of cell growth and the current viable cell concentration. As a first approximation in this study, we choose the Luedeking–Piret model to describe the kinetics of product formation in the recombinant cell cultures (Eq. 5). This model is based on the premise that the rate of product synthesis can be a function of both cellular density ([X]) and the rate of cell growth (r_x_). We also assumed that a Luedeking–Piret model could describe the kinetics of mAb throughout the entire duration of the cell culture.

To determine values for the parameters of the model, we followed a straight-forward approach. The values of α and β were chosen to fit several experimental data sets of cell concentration and mAb production profiles. We fit polynomial models to the experimental profiles of cell density versus time observed in r-CHO cell cultures. From these polynomials, values of the cell concentration ([X]) and the rate of cell growth (r_x_) at any given time were calculated. We used these values ([X], r_x_) together with α and β sets to numerically integrate the Luedeking–Piret equation (see Eq. 6). The best fit (shown as a dashed line in Fig. 4) was achieved using the values of α = 7.65 × 10^−7^ µg/cell/h, and β = 7.68 × 10^−8^ µg/cell. These α and β values can be used to accurately reproduce data obtained in experiments where the initial substrate concentration ranges from 2.4 to 4.5 g/L (undiluted medium).

The absence of both internal stirring and sparging in shake flasks has been shown to generate culture conditions substantially different from those in bioreactors (Sieck et al. 2013). Duplicate batch processes were performed with recombinant CHO cell cultures in 1.6-L (1.0-L w/v) bioreactors at an undiluted medium concentration. Figure 3b shows the cell and mAb profiles for a representative batch process of approximately 250 h, with a peak cell density of 4 × 10^6^ cells/mL on day 7, and a final mAb titer of 40 mg/L. As compared to the cell profiles observed in shake flasks, there was a lag-phase up to 3 days, which resulted in longer processes.

In this set of bioreactor experiments, the mAb concentration time series, was well described by a Luedeking–Piret model. The values of α = 7.65 × 10^−7^ µg/cell/h and β = 7.68 × 10^−8^ µg/cell that produced the best fit for shake flask batch experiments, fit the experimental data well (Fig. 4b).

We also validated the use of this parameter set in fed-batch cultures in instrumented bioreactors, conducted under a dual phase culture strategy: (a) a first period of cell growth at 37 °C followed by (b) a mAb production stage at 33 °C. As before mentioned, this protocol more accurately represents the standard practice for recombinant CHO cell cultures. Figure 4c shows the cell and mAb profiles for a representative fed-batch experiment conducted in a 1.5-L instrumented bioreactor. In this case, process duration was approximately 450 h, with a peak cell density of 6 × 10^6^ cells/mL on day 8 and a final mAb titer of 90 mg/L. The culture was set to 37 °C for the first 7 days of culture. At day 7, the temperature set point was decreased to 33 °C.

As a direct result of the temperature shift and fed batch protocol, the cell production rate at the late exponential growth phase was reduced, and a high viable cell density was maintained for 5 days, resulting in a prolonged culture. We observed a shift from peak cell density to peak antibody production. There was also a twofold increase in mAb production (from 40 to 90 mg/L). The Luedeking–Piret model previously tested for batch processes (in shake flasks and bioreactors) fit the experimental data (shown as a dashed line in Fig. 3c), while maintaining the same Luedeking–Piret constants.

### Monod and Luedeking–Piret parameters at different temperatures

For the clone used in this study, we found that higher final mAb titers can be obtained by setting the culture temperature at 33 °C during exponential growth and later decreasing it to 31 °C or by maintaining the temperature at 33 °C during the entire culture. The Monod parameters that we reported in a previous section are valid for exponential growth at 33 °C. The proposed parameter values for the Luedeking–Piret model fit reasonably well with several independent experimental data sets in the range of 31–33 °C. However, different protocols are used to culture CHO cells in commercial practice and academic work. Not infrequently, a temperature of 37 °C is used during the exponential growth period normally corresponding to the first 7 days of culture. We used three independent sets of data from the batch culture experiment of r-CHO cells at 37 °C to adjust the Monod and Luedeking–Piret values during exponential growth at this temperature. The differential material balance equations for cell growth, substrate consumption, and mAb formation were numerically integrated. The parameter values were determined for growth, substrate consumption, and product formation for cultures at 33 °C as an initial guess. Results from the numerical integration were compared to [X], [S], and [P] experimental time series. We observed that, by modifying the values of μ_max_ and Y_x/s_, an appropriate fit was achieved with respect to the experimental time series {t,[X],[S]} of several experimental data sets at 37 °C. The values of K_s_ and [S]_t_ calculated from experiments at 33 °C were used to properly fit the data at 37 °C. However, for mAb production in batch experiments at 37 °C, we were unable to simulate different experimental sets using the same α and β values from the Luedeking–Piret model. The time series of [mAb], [X], and [S] versus time can be reasonably described during batch cultivation experiments at 37 °C by keeping the value of α = 7.65 × 10^−7^ (the same values calculated from experiments at 33 °C) and setting β in the range of 1.30–3.50 × 10^−8^ cell/µg. The inclusion of an inhibitory effect of lactate concentrations in mAb production should improve the performance of the model at T = 37 °C. In Table 2, we summarized all the kinetic parameters determined for naive and recombinant cultures at 33 and 37 °C.

## Conclusions

In summary, we studied the kinetics of cell growth, glucose consumption, and product formation in mAb-producing recombinant CHO cells at 33 °C, and compared them to their naive counterparts. It was found that, for these specific cell lines, simple kinetic models could explain cellular growth, glucose consumption, and product formation. For the cellular growth, a Monod-type model was proposed with a modification to account for a threshold glucose concentration of 0.6 g/L. The overall rate of glucose consumption can be properly described using a constant cellular yield. Product formation was found to be related to the rate of cell growth and the cellular density itself through a Luedeking–Piret-type model. We determined the Luedeking–Piret-type constants for batch cell cultures conducted in shake flasks and stirred bioreactors. The set of kinetic parameters calculated from batch cultures properly described growth, glucose consumption, and mAb formation in fed-batch processes.
